# Pulmonary actinomycosis mimicking a lung metastasis from esophageal cancer; a case report

**DOI:** 10.1186/s12890-018-0602-z

**Published:** 2018-02-27

**Authors:** Munemasa Nagao, Akihisa Fukuda, Takeshi Matsumura, Toshiyuki Kimura, Hiroshi Seno

**Affiliations:** 1 Department of Gastroenterology and Hepatology, Graduate School of Medicine, Kyoto University, 54 Kawahara-cho, Syogoin, Sakyo-ku, Kyoto, 606-8507 Japan; 2 Department of Gastroenterology and Hepatology, Hyogo Prefectural Amagasaki General Medical Center, Amagasaki, Japan

**Keywords:** Actinomycosis, Esophageal cancer, Infection

## Abstract

**Background:**

Actinomycosis is a rare bacterial infection caused by *Actinomyces*. The symptom of actinomycosis is nonspecific and radiological images present as a slow-progressive mass lesion similarly to malignancies. Thus, it is difficult to distinguish pulmonary actinomycosis from malignancies.

**Case presentation:**

A 74-year-old male who had esophageal cancer and a pulmonary mass that was positive for ^18^F–fluorodeoxyglucose positron emission tomography/computed tomography was initially diagnosed with esophageal cancer with a lung metastasis because he was asymptomatic. However, aspiration of pleural effusion revealed that the pulmonary lesion was actinomycosis.

**Conclusion:**

We present a case of pulmonary actinomycosis mimicking a lung metastasis from esophageal cancer. Diagnosis of asymptomatic pulmonary actinomycosis is difficult, and needle aspiration could be useful for a definitive diagnosis of pulmonary actinomycosis.

## Background

Actinomycosis is a rare chronic granulomatous infection caused by *Actinomyces* species. *Actinomyces* are facultative anaerobic Gram-positive bacteria [1]. The symptom of actinomycosis is nonspecific and similar to those of other chronic suppurative chest diseases. On computed tomography (CT) and ^18^F–fluorodeoxyglucose positron emission tomography/computed tomography (PET/CT), pulmonary actinomycosis presents as a slow-progressive mass lesion similarly to malignancies. Non-specific symptoms and radiological images make diagnosis of pulmonary actinomycosis difficult [2, 3]. In previous case reports, pulmonary actinomycoses were occasionally resected as pulmonary neoplasms because of its difficult diagnosis [4]. Here we report a case of pulmonary actinomycosis mimicking a lung metastasis from esophageal cancer.

## Case presentation

**Fig. 1:**
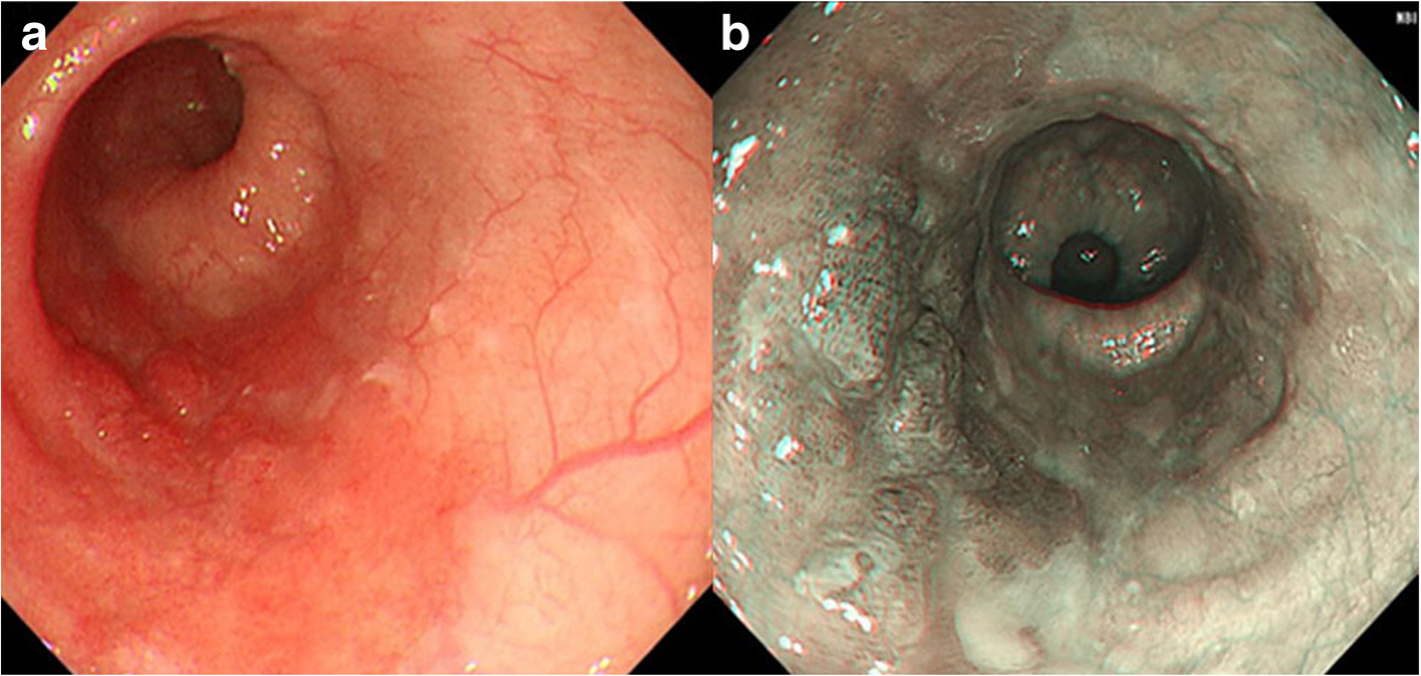
Images of esophago-gastro-duodenoscopy (EGD). **a** White Light Imaging. EGD showed a 0-IIa lesion with granular nodules on the lower thoracic esophagus. **b** Narrow Band Imaging. The lesion showing the brownish area was predicted to be squamous cell carcinoma

**Fig. 2:**
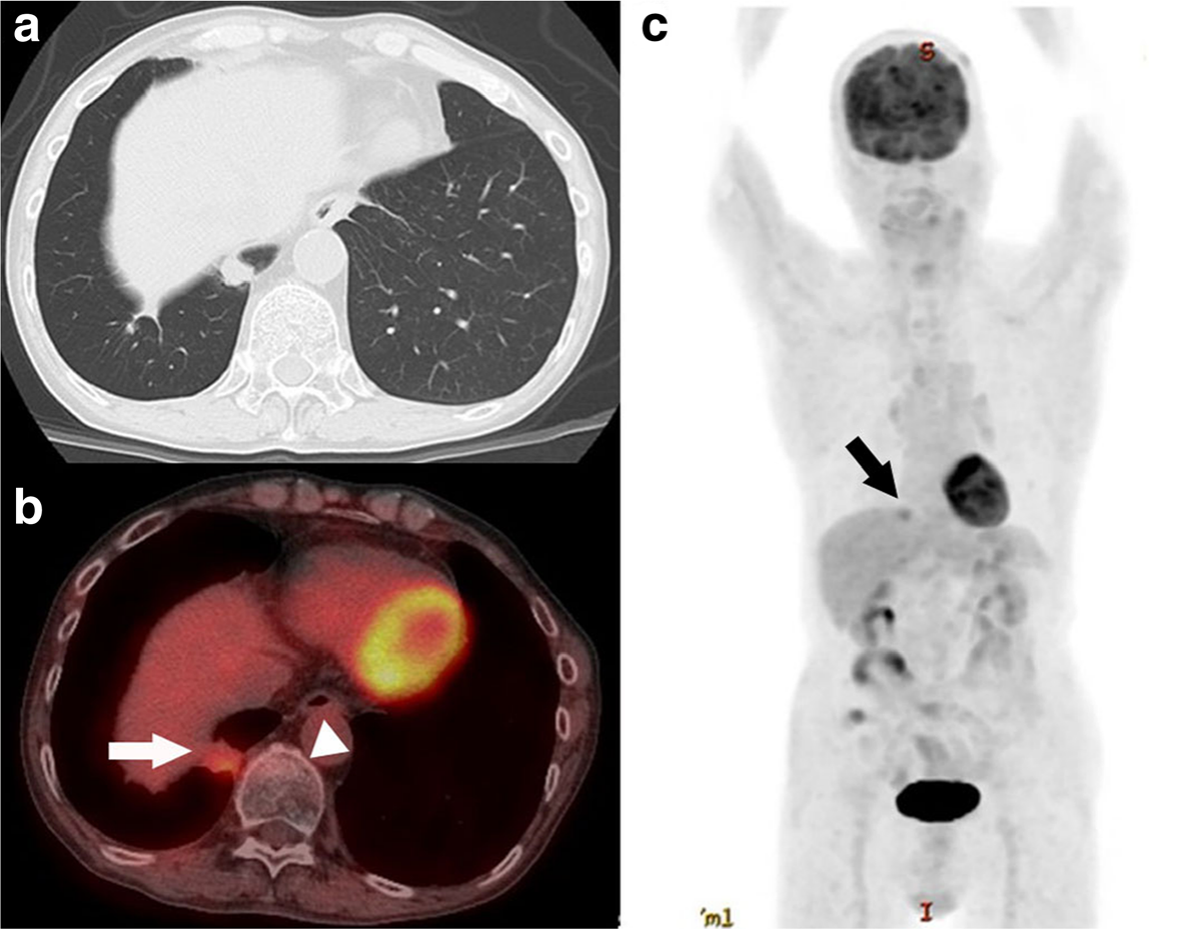
Images of computed tomography (CT) and Positron emission tomography (PET) /CT before admission to the hospital. **a** CT showing a pulmonary mass in the right lower lobe. **b** and **c**: PET/CT showed the pulmonary mass had maximal standardized uptake value (SUVmax) of 3.88 and the lower thoracic esophagus had SUVmax of 2.37

**Fig. 3:**
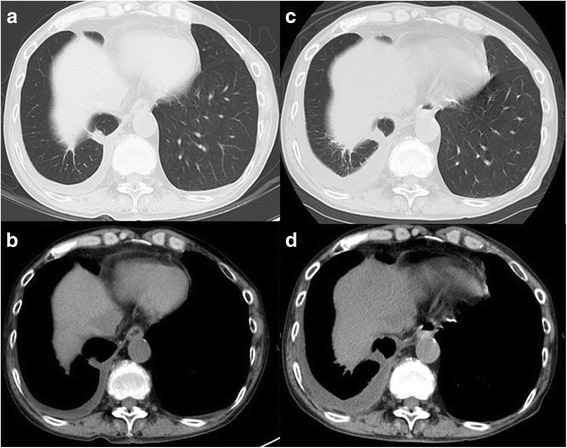
Images of CT after admission to the hospital. **a** and **b** on the day 8, (**c**) and (**d**) on the day 14. CT revealed an increased amount of pleural effusion

**Fig. 4:**
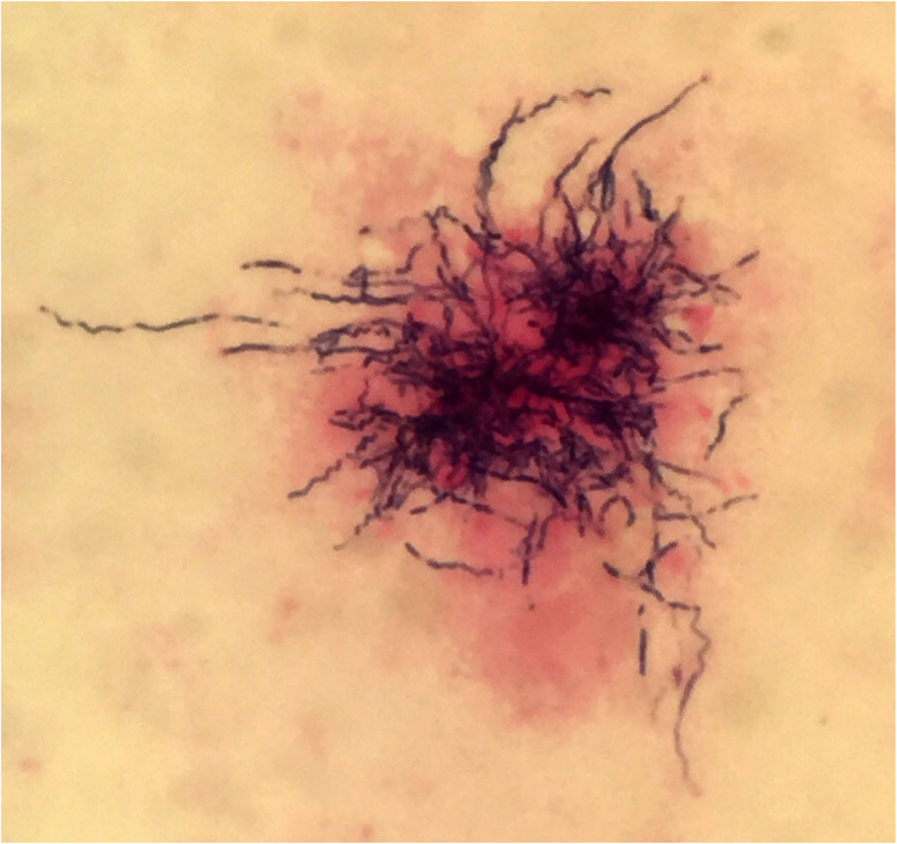
Gram stain of aspirate from pleural effusion. Gram stain of aspirate from pulmonary mass revealed *Actinomyces israelii* as bronching, gram-positive filaments

**Fig. 5:**
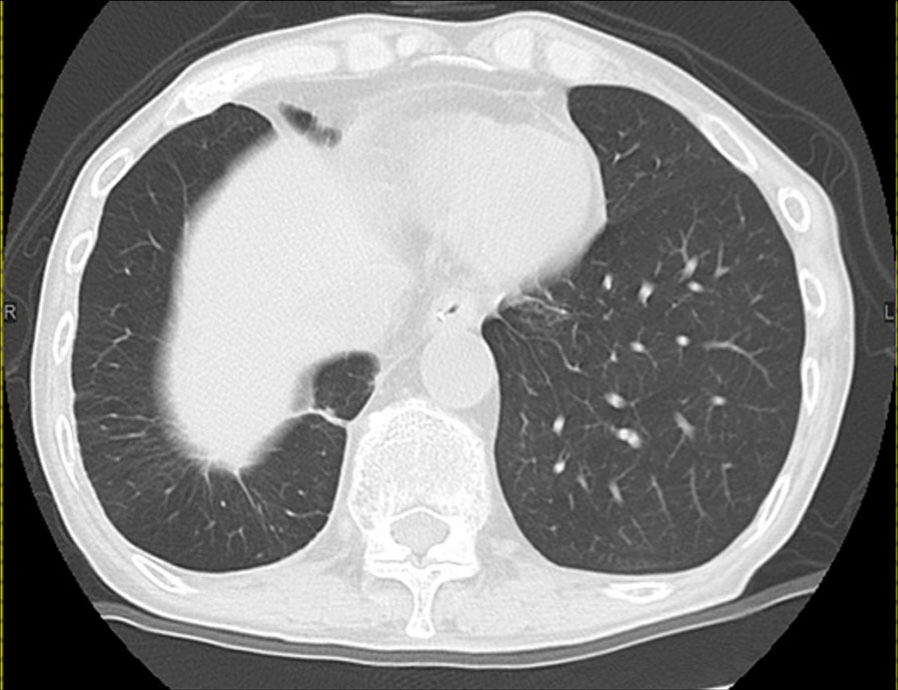
Image of chest CT 6 months after the treatment with antibiotics. The pulmonary actinomycosis disappeared

A 74-year-old male was admitted to our hospital for the treatment of esophageal cancer. The patient had no symptoms with normal temperature at the time of admission. His past medical history included diabetes mellitus for 24 years. He underwent endoscopic submucosal dissection for early gastric cancer 4 years ago and video-assisted thoracic surgery for pulmonary adenocarcinoma in the right upper lobe 5 years ago. He occupation was making metal molds. He was ex-smoker: Brinkman index was 980. Laboratory studies showed white blood cells of 10.0 × 10^3^/L, C-reactive protein of 6.07 mg/dL, hemoglobin A1c test of 8.8%, fasting blood sugar of 237 mg/dL on admission. Serum tumor markers, including CEA, CA19–9, SCC, CYFRA, were unremarkable. He underwent esophago-gastro-duodenoscopy for follow-up after endoscopic submucosal dissection for early gastric cancer. The upper endoscopy showed a 0-IIa lesion with relatively large granular nodules on the lower thoracic esophagus (Fig. 1). The pathologic assessment of the biopsy revealed squamous cell carcinoma of the esophagus. The esophageal 0-IIa lesion with relatively large granular nodules let us consider the depth of esophageal cancer was deeper than M3/SM1. Chest X-Ray was normal, however, chest CT revealed a 1.3 cm × 0.9 cm pulmonary mass in the lower lobe of the right lung a month before admission (Fig. 2a). PET/CT showed the pulmonary mass had maximal standardized uptake value (SUVmax) of 3.88 (arrow) and part of the lower thoracic esophagus had SUVmax of 2.37 (arrowhead) 2 weeks before admission (Fig. 2b and c). Because the image of pulmonary mass had not changed between CT and PET/CT for 2 weeks and because he had no fever, even though he had inflammation reaction on laboratory studies, pulmonary mass was considered to be unlikely due to infection. Thus, he was initially diagnosed with esophageal cancer with a pulmonary metastasis or recurrence of a pulmonary adenocarcinoma. Considering the both possibilities, we started chemotherapy with cisplatin (CDDP) 70 mg/m^2^ day1 plus 5-fluorouracil (5-FU) 700 mg/m^2^ day1–4 on the day 4 after admission. On the day 8, CT revealed a slight pleural effusion in the right side (Fig. 3a and b). On the day 9, to rule out carcinomatous pleurisy, an ultrasound-guided aspiration of pleural effusion was performed. The effusion was serous and contained no malignant cells. After the aspiration, the patient got a high fever. Laboratory test showed white blood cells of 18.6 × 103/L (90.8% of Neutrophil), CRP of 25.87 mg/dL. Considering that fever was due to respiratory infection after the aspiration, intravenous Sulbactam/Ampicillin (6 g/day) was administered. On the day14, his fever was persistent and he also had a cough. Because CT revealed an increased amount of pleural effusion (Fig. 3c and d), we considered the mass was lung abscess. By the second ultrasound-guided aspiration of the pleural effusion, we got a 50 ml of white suppurative effusion. The effusion turned out to contain *Actinomyces israelii* (Fig. 4), and proved pulmonary mass-like lesion was pulmonary actinomycosis. The patient received intravenous penicillin G (24 million units/day) for 4 weeks, followed by oral amoxicillin 2250 mg/day for 6 months. The patient received four courses of chemotherapy (CDDP/5-FU) and curative radiotherapy (1.8Gy/total 50.4Gy) as esophageal cancer deeper than M3/SM1. The pulmonary actinomycosis disappeared six months after the treatment with antibiotics (Fig. 5). After the chemoradiation therapy without any complications, the esophageal cancer was in complete remission.

## Discussion

Actinomycosis is a rare and chronic bacterial infection caused by *Actinomyces* species. *Actinomyces israelii* is one of the *Actinomyces* species. *Actinomyces* can colonize the cervicofacial, thoracic, abdominal, pelvic lesions, skin, brain and other organs. Pulmonary actinomycosis accounts for 15% of all actinomycoses [5], and is mainly caused by aspiration of oral saphrophytes into respiratory tracts [3]. As actinomycosis can develop after dental care or aspiration, careful history taking is necessary for old ages. Sputum examination and culture is difficult to detect pathogenic bacteria, because *Actinomyces* are anaerobic and exist in normal oral cavity. Clinical symptoms of pulmonary actinomycosis include chest pain, chronic cough, hemoptysis, fever and chill [1], but actinomycosis frequently has no symptoms. Nonspecific symptoms or no symptoms make a definitive diagnosis difficult. Radiologically, pulmonary actinomycosis presents as a slow-progressive consolidation or mass-like shadow. Pulmonary actinomycosis often has central attenuation area or dilation bronchiole shadow on CT. Central low attenuation area consists of one or more, round or oval low attenuation area at the center of the shadow [6], which is characteristic of pulmonary actinomycosis. ^18^F–FDG PET/CT is useful for detecting malignancies including metastases in the whole body, but various inflammations and infections also have FDG uptake [2, 3]. The interpretation of PET/CT finding needs to be careful with primary malignant lesions. Even experienced physicians could delay diagnosis or misdiagnose with tuberculosis or lung cancer [7]. For a definitive diagnosis, biopsy is often required. However, success of CT-guided, ultrasound-guided, or bronchial biopsy is dependent on the location of pulmonary mass [8]. Lung resection was occasionally performed as a diagnosis of pulmonary neoplasm and pulmonary actinomycosis was diagnosed by the pathology of the resected lung specimens [9, 10]. Regarding to the therapy of actinomycoces, almost all *Actinomyces* is sensitive to penicillin. For *Actinomyces* forming chronic granulation, penicillin in high doses is usually needed for long duration (6–12 months) to prevent relapse. Risk factors associated with the acquisition of *Actinomyces* are diabetes mellitus, male sex, and immunosuppressive condition such as a use of steroid and alcoholism [7]. In this case, the patient’s long-term diabetes mellitus is considered to be a risk factor for infection of *Actinomyces*. The patient had primary esophageal cancer and past history of primary pulmonary adenocarcinoma, which made a diagnosis difficult. However, a high fever after starting chemotherapy gave us a clue of the infection and prompted us to perform needle aspiration of the pleural effusion.

## Conclusion

Even though rare, pulmonary actinomycosis should be included in differential diagnosis of PET/CT positive pulmonary mass without any symptoms. Especially, if a patient has other primary malignant lesions, it is difficult to distinguish pulmonary actinomycosis from a lung metastasis. If available, needle aspiration could be useful for a definitive diagnosis of pulmonary actinomycosis.
